# The change in nutritional status is related to cardiovascular events in patients with pacemaker implantation: A 4-year follow-up study

**DOI:** 10.3389/fnut.2022.986731

**Published:** 2022-09-02

**Authors:** Kaijing Wang, Liyou Lian, Chengpu Chen, Meiling Wang, Chen Chen, Xiang Hu

**Affiliations:** ^1^Department of Ultrasound, The First Affiliated Hospital of Wenzhou Medical University, Wenzhou, China; ^2^Department of Cardiology, The First Affiliated Hospital of Wenzhou Medical University, Wenzhou, China; ^3^The Key Lab of Cardiovascular Disease, Science and Technology of Wenzhou, Wenzhou, China; ^4^Key Laboratory of Clinical Laboratory Diagnosis and Translational Research of Zhejiang Province, Department of Endocrine and Metabolic Diseases, The First Affiliated Hospital of Wenzhou Medical University, Wenzhou, China

**Keywords:** pacemaker implantation, prognostic nutritional index, geriatric nutritional risk index, nutritional status, prognosis

## Abstract

**Background:**

The aim of our study was to evaluate changes in nutritional status as measured by the prognostic nutritional index (PNI) and geriatric nutritional risk index (GNRI) scores, and their abilities to predict clinical prognosis in patients with pacemaker implantation (PMI).

**Methods:**

A total of 595 patients who underwent permanent PMI from January 2011 to December 2020 were included. PNI and GNRI scores were separately calculated at the beginning day of PMI operation and at the end of 12-month follow-up, and their net changes (Δ) were calculated by PNI or GNRI scores at follow-up minus the corresponding scores on admission. The cohort patients were divided into low risk of malnutritional status (ΔPNI or ΔGNRI scores ≥ 0) and high risk of malnutritional status (ΔPNI or ΔGNRI scores < 0) groups. Primary outcome measure was a composite major adverse cardiovascular event (MCE), defined as heart failure hospitalization (HFH), myocardial infarction (MI), stroke, or death from any cause, presented as hazard ratios (HR) with 95% confidence intervals (CI) calculated by MCE in the crude or multivariate-adjusted Cox Proportional Hazards models. Receiver operating characteristic (ROC) curve analysis was used to compare the differential ability to predict incident MCEs betweenΔPNI andΔGNRI scores.

**Results:**

In total, 16% of patients developed the MCE during the follow-up. The cumulative event rates determined by Kaplan–Meier analysis were significantly higher in the high risk of malnutritional patients compared to the low risk of malnutritional patients (*P* < 0.05). Adjusted multivariate analysis showed that decreased PNI scores (HR: 2.228, 95% CI: 1.482–3.350) and decreased GNRI scores (HR: 2.178, 95% CI: 1.439–3.295) were independently associated with favorable outcomes. ROC curve analysis revealed an area under curve (AUC) of 0.586 forΔPNI scores and AUC of 0.592 for ΔGNRI scores, but their predictive abilities were not statistically different.

**Conclusion:**

Either positive change of PNI or GNRI scores were associated with reduced risk of MCEs in patients with PMI, and they have similar ability to predict clinical cardiometabolic risk. Additional enhancing nutritional status during follow-up may help to prevent unfavorable prognosis in clinical practices.

## Introduction

Pacemaker implantation (PMI) is an effective treatment for patients who continue to have symptoms of heart failure (HF) while receiving adequate medical care (1). It is well-established that PMI can help reduce the rate of HF hospitalization (HFH) and atrial fibrillation (AF) in pacing-dependent groups (2). However, some individuals are unable to benefit from this therapy for a variety of reasons, resulting in suboptimal outcomes (3).

Malnutrition is a common comorbidity in elderly individuals and is related with a worse prognosis (4, 5). Additionally, PMI recipients have grown older and more complex, with numerous comorbidities (6). Thus, the nutritional status in this group needs additional monitoring. Several studies have demonstrated an association between a single nutritional indicator (7–9), such as albumin, and poor outcomes in individuals with chronic HF. However, to evaluate the nutritional status, more complex and objective indices are needed because single indicator might be inflected by many external factors. Take albumin for instance. Albumin (and other serum proteins generated by the liver) is altered not just by nutritional state, but also by inflammation and infection, limiting its utility in acutely unwell individuals. Additionally, albumin’s extended half-life reduces its utility for monitoring short-term changes in both calorie and protein intakes. Several nutritional screening measures have been presented for the purpose of assessing malnutrition and its long-term prognosis (10).

The prognostic nutritional index (PNI) score is generated using blood albumin and lymphocyte counts to describe the immunological nutritional status of surgical patients and to estimate the probability of developing a complication (11). Another nutrition screening measure frequently used in individuals with HF is the geriatric nutritional risk index (GNRI) score (12). These two indices can be determined using only simple blood biomarkers. The nutritional status might constantly change; therefore, the prognostic value of PNI and GNRI scores for adverse clinical outcomes could also change over time. A recent study (13) reported that change in PNI score was associated with clinical outcomes in patients with cardiac resynchronization therapy (CRT)-device. Another study (14) proved that remaining at nutritional risk in transcatheter aortic valve replacement group resulted in an increased risk of mortality and HFH. Thus, positive changes in the PNI and GNRI scores during follow-up following PMI may be associated with improved cardiac function and subsequent clinical outcomes; however, few studies regarding associations of changes in nutritional status and clinical outcomes in patients with pacemakers have been reported. Therefore, present study evaluated changes in nutritional status as measured by the PNI and GNRI scores, and their abilities to predict clinical prognosis in patients with PMI.

## Materials and methods

### Study subjects

This was a retrospective analysis of patients who had successful PMI at the First Affiliated Hospital of Wenzhou Medical University between January 2011 and December 2020 with complete follow-up data. Blood collection, 12-lead electrocardiography (ECG), and echocardiography were used to evaluate the clinical status prior to PMI. The indications for pacemakers were recorded [atrioventricular block (AVB), sick sinus syndrome (SSS), and atrial fibrillation (AF) with bradycardia, left bundle branch block (LBBB) with HF, AF with atrioventricular node ablation (ANVA)]. All patients were evaluated thoroughly 1 year after implantation, including blood sample analysis and echocardiography. Patients who were lost to follow-up or lacked the necessary data were omitted from this study.

The net changes (Δ) were calculated by PNI or GNRI scores at follow-up minus the corresponding scores on admission. The cohort patients were divided into low risk of malnutritional status (ΔPNI or ΔGNRI scores ≥ 0) and high risk of malnutritional status (ΔPNI or ΔGNRI scores < 0) groups.

We investigated the influence of ΔPNI and ΔGNR scores following PMI on laboratory data, cardiac function, and significant adverse cardiovascular events (MCEs).

The PNI and GNRI scores are nutritional risk indicators that are associated with the severity of malnutrition and mortality rate in hospitalized patients. They were calculated as following formula:

PNI scores = serum albumin (g/L) + 0.005 × total lymphocyte count (per mm^3^)

GNRI scores = 1.489 × serum albumin level (g/L) + 41.7 × [actual bodyweight (ABW)/ideal bodyweight (IBW)]

Ideal body weight was calculated using the Lorentz formula (12): height (cm) –100– {[height (cm) –150]/4} for males or height (cm) –100– ([height (cm) –150]/2.5) for females. If the ratio of actual body weight (kg) to ideal body weight (kg) was ≥ 1, the assigned value was 1, as previously published (14).

### Data collection

Baseline information was investigated, including BMI, smoking and drinking history and concurrent disease. The echocardiographic parameters included the left ventricular end-diastolic diameter (LVEDD), left atrial diameters (LAD), LV ejection fraction (LVEF), mitral regurgitation (MR), and tricuspid regurgitation (TR). Laboratory data included hemoglobin, total lymphocyte count, albumin, triglycerides (TG), total cholesterol (TC), high density lipoprotein (HDL), low density lipoprotein (LDL), aspartate aminotransferase (AST), white blood cell (WBC), hemoglobin (Hb), platelets (PLT), and the PNI and GNRI scores.

### Identification of major adverse cardiovascular event during the follow-up

The follow-up of adverse events continued until December 2021. The MCE was readmission to the hospital due to worsening HF, myocardial infarction, stroke, or death from any cause. Only the first occurrence was included in the analysis for patients who experienced two or more events. The diagnosis of worsening HF was an unplanned hospital admission or an urgent HF visit resulting in intravenous therapy for HF. Myocardial infarction is used when the evidence of myocardial injury (defined as an elevation of cardiac troponin values with at least one value above the 99th percentile upper reference limit) with necrosis in a clinical situation consistent with myocardial ischemia (15). The status and/or dates of death of all patients were gathered from the patients’ medical records or attending physicians at the patient’s referring hospital. All patients were followed in this study. The duration of survival was calculated from the date of PMI to the date of readmission, death, or last follow-up.

### Statistical analyses

Data were presented as percentages for categorical variables and mean ± standard deviation (SD) or median and interquartile range (IQR) for continuous variables. Group differences were evaluated using Student *t*-tests or Mann–Whitney *U* tests for continuous variables and chi-square or Fisher exact tests for categorical variables. The crude or multivariate-adjusted Cox Proportional Hazards models were used to estimate the hazard ratios (HR) and 95% confidence intervals (CI) calculated by MCE without or with the additional adjustments including baseline sociodemographic parameters, lifestyle factors, prevalent diseases, with the low risk of malnutrition group as a reference category. The cumulative incidence curve of adverse events was plotted via the Kaplan-Meier method, with statistical significance examined by the log-rank test. Receiver operating characteristic (ROC) curve analysis was used to compare the difference of the changes in PNI and GNRI scores in the ability of predicting prognosis. ROC curves were generated for the changes of PNI and GNRI scores, and areas under the curve (AUCs), cut-of values, sensitivities, and specificities were calculated. All analyses were performed using R V.4.1.3 or IBM SPSS version 26.0 (IBM Corp., Armonk, NY, United States) and tests were two-sided and *P* < 0.05 was considered statistically significant.

## Results

### Patient characteristics

Between January 2010 and December 2020, our study enrolled 595 individuals. The basic information was detailed in Table 1. The median age was 70 (interquartile range 63–77) years and 63% of the population were men. The median PNI and GNRI scores at baseline were 46.9 (interquartile range 43.0–50.3), 102.1 (interquartile range 96.2–108.5), respectively. The BMI was 23.7 (interquartile range 21.9–26.2) kg/m^2^. The hypertension, DM, ICM accounted for 57, 23, and 26%, respectively. In our study, more patients were in high risk of malnutritional status either measured by PNI or GNRI (69 and 75%, respectively).

**TABLE 1:** Characteristics of the study population.

Variable	Total	ΔPNI			ΔGNRI		
			
		Decreasing nutritional status	Increasing nutritional status	*P*	Decreasing nutritional status	Increasing nutritional status	*P*
Participants (n, %)	595 (100)	412 (69)	183 (31)		424 (71)	171 (29)	
Men (n, %)	375 (63)	259 (63)	116 (63)	0.903	271 (64)	104 (62)	0.479
Age (y)	70 (63–77)	69 (63–76)	71 (63–77)	0.526	69 (62–76)	72 (64–78)	0.058
BMI (kg/m^2^)	23.7 (21.9–26.2)	23.7 (22.0–26.1)	23.5 (21.3–26.3)	0.286	23.7 (22.0–26.2)	23.7 (21.3–26.3)	0.273
Follow-up periods (years)	2.0 (1.0–4.0)	2.0 (1.0–4.0)	2.0 (1.0–3.8)	0.017	2.0 (1.0–3.9)	2.0 (1.0–4.0)	0.722
**Lifestyle risk factors (n, %)**							
Smoking history	164 (28)	105 (25)	59 (32)	0.089	115 (27)	49 (29)	0.705
Drinking history	154 (26)	104 (25)	50 (27)	0.593	110 (26)	44 (26)	0.957
**Concomitant disease (n, %)**							
Hypertension	342 (57)	242 (59)	100 (55)	0.351	247 (58)	95 (56)	0.547
DM	136 (23)	102 (25)	34 (19)	0.098	107 (25)	29 (17)	0.030
ICM	152 (26)	104 (25)	48 (26)	0.799	109 (26)	43 (25)	0.887
**Echocardiography**							
LVEF (%)	61.4 (39.0–68.6)	61.5 (42.0–68.6)	60.9 (37.0–67.4)	0.226	62.2 (41.3–69.4)	60.6 (37.0–66.9)	0.056
LAD (mm)	47.0 (42.0–52.0)	47.0 (42.0–52.0)	46.0 (41.0–52.0)	0.627	47.0 (42.0–52.0)	46.0 (41.0–52.0)	0.888
LVEDD (mm)	54.0 (49.0–60.0)	54.0 (49.0–60.0)	55.0 (49.0–60.0)	0.408	54.0 (49.0–60.0)	55.0 (49.0–62.0)	0.188
MR	1.0 (1.0–2.0)	1.0 (0.6–2.0)	1.0 (1.0–2.0)	0.033	1.0 (1.0–2.0)	1.0 (1.0–2.0)	0.018
TR	1.0 (0.0–2.0)	1.0 (0.0–2.0)	1.0 (0.5–2.0)	0.998	1.0 (0.0–2.0)	1.0 (0.0–2.0)	0.896
**Laboratory metrics**							
TC (mmol/L)	4.1 (3.4–4.8)	4.0 (3.4–4.7)	4.2 (3.5–5.0)	0.065	4.1 (3.4–4.8)	4.1 (3.4–5.0)	0.408
TG (mmol/L)	1.2 (0.9–1.7)	1.2 (0.9–1.6)	1.2 (0.9–1.8)	0.461	1.2 (0.9–1.6)	1.3 (0.9–1.8)	0.307
HDL-c (mmol/L)	1.0 (0.9–1.2)	1.0 (0.8–1.1)	1.1 (0.9–1.3)	0.000	1.0 (0.8–1.2)	1.1 (0.9–1.3)	0.001
LDL-c (mmol/L)	2.2 (1.7–2.8)	2.2 (1.7–2.8)	2.3 (1.7–2.9)	0.611	2.2 (1.7–2.8)	2.3 (1.7–2.9)	0.899
AST (U/L)	24 (20–31)	23 (20–31)	25 (21–31)	0.158	24 (20–31)	24 (20–31)	0.581
WBC (10^9^/L)	6.1 (5.0–7.4)	6.1 (5.0–7.4)	6.1 (5.1–7.4)	0.849	6.1 (5.1–7.5)	6.0 (4.8–7.2)	0.268
PLT (10^9^/L)	186 (153–225)	185 (153–227)	188 (150–223)	0.830	185 (153–227)	188 (148–223)	0.602
Hb (g/L)	130 (119–142)	129 (119–140)	130 (120–142)	0.238	130 (120–142)	129 (118–141)	0.620
**Types of the pacemakers**							
Single-chamber	13 (2.2)	7 (3.8)	6 (1.5)	0.129	7 (4.1)	6 (1.4)	0.087
Dual-chamber	334 (56.1)	97 (53.0)	237 (57.5)	0.350	85 (49.7)	249 (58.7)	0.055
CRT-P	97 (16.3)	28 (15.3)	69 (16.7)	0.748	33 (19.3)	64 (15.1)	0.257
CRT-D	133 (22.4)	45 (24.6)	88 (21.4)	0.443	40 (23.4)	93 (21.9)	0.781
Dual-ICD	18 (3.0)	6 (3.3)	12 (2.9)	0.998	93 (21.9)	12 (2.8)	0.862
**Pacing indications**							
AVB	144 (24.2)	36 (19.7)	108 (26.2)	0.106	35 (20.5)	109 (25.7)	0.213
SSS	125 (21.0)	34 (18.6)	91 (22.1)	0.390	28 (16.4)	97 (22.9)	0.099
AF with bradycardia	104 (17.5)	34 (18.6)	70 (17.0)	0.723	31 (18.1)	73 (17.2)	0.884
LBBB with HF	63 (15.3)	26 (14.2)	89 (15.0)	0.828	51 (29.8)	82 (19.3)	0.998
AVNA	80 (19.4)	53 (29.0)	133 (22.4)	0.013	26 (15.2)	63 (14.9)	0.008

Data were presented as percentages for categorical variables and mean ± standard deviation (SD) or median and interquartile range (IQR) for continuous variables. Group differences were evaluated using Student t-tests or Mann–Whitney U tests for continuous variables and chi-square or Fisher exact tests for categorical variables.

PNI, prognostic nutritional index; GNRI, geriatric nutritional risk index; BMI, body mass index; DM, diabetes mellitus; ICM, ischemic cardiomyopathy; LVEF, left ventricular ejection fraction; LAD, left atrial diameter; LVEDD, left ventricular end-diastolic diameter; MR, mitral regurgitation; TR, tricuspid regurgitation; TC, total cholesterol; TG, triglyceride; HDL-c, high-density lipoprotein- cholesterol; LDL-c, low-density lipoprotein- cholesterol; AST, aspartate transaminase; WBC, white blood cell; PLT, platelet; Hb, hemoglobin; CRT-P, cardiac resynchronization therapy-pacemaker; CRT-D, cardiac resynchronization therapy- defibrillator; dual-ICD, dual-implantable cardioverter defibrillator; AVB, atrioventricular block; SSS, sick sinus syndrome; AF, atrial fibrillation; LBBB, left bundle branch block; HF, heart failure; AVNA, atrial fibrillation with atrioventricular node ablation.

The types of the pacemakers were single-chamber (2%), dual-chamber (56%), cardiac-resynchronization therapy-pacemaker (CRT-P, 16%), CRT-defibrillator (22%), dual-implantable cardioverter defibrillator (dual-ICD, 3%). The most common reasons for pacing were AVB, AF with AVNA, and SSS, accounting 24, 19, and 21%, respectively. AF with slow ventricular rate was 17% and LBBB with HF was 18%.

### Nutritional status and major adverse cardiovascular event risk

**FIGURE 1:**
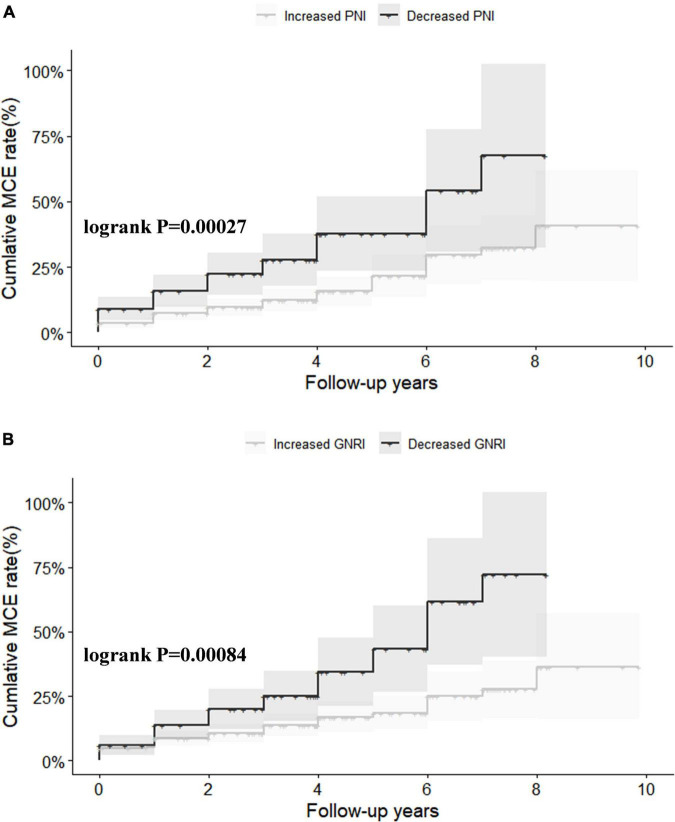
Kaplan–Meier survival analysis for major adverse cardiovascular event (MCE) stratified by ΔPNI **(A)** and ΔGNRI **(B)**. PNI, prognostic nutritional index; GNRI, geriatric nutritional risk index.

All clinical follow-up data were obtained. The median follow-up period was 2.0 (1.0–4.0) years. During the follow-up, 95 (16%) MCEs were identified. Figure 1 shows Kaplan–Meier curves for MCE among patients stratified by ΔPNI and ΔGNRI scores. In high risk of malnutritional status groups, the cumulative incidence of MCEs clearly increased (log-rank test, *P* < 0.0001 each).

Table 2 shows Cox proportional hazard analyses for MCEs. On unadjusted Cox modeling, rates of MCEs rose progressively with low risk of malnutrition groups, even after adjusting for other risk factors (PNI scores < 0: HR: 2.228, 95% CI 1.482–3.350, *P* < 0.000; GNRI scores < 0: HR: 2.178, 95% CI 1.439–3.295, *P* < 0.000).

**TABLE 2:** The association of ΔPNI, ΔGNRI with major adverse cardiovascular event (MCE) risk.

	Association between the ΔPNI and MCE risk			Association between the ΔGNRI and MCE risk		
		
	HR	95%CI	*P*	HR	95%CI	*P*
Model 1	2.056	1.372–3.082	0.000	1.943	1.295–2.914	0.001
Model 2	2.045	1.364–3.065	0.001	1.913	1.271–2.879	0.002
Model 3	2.055	1.370–3.083	0.000	1.915	1.272–2.884	0.002
Model 4	2.228	1.482–3.350	0.000	2.178	1.439–3.295	0.000

We performed Cox Proportional Hazards models of patients stratified according to PNI and GNRI improved or decreased, respectively.

Model 1: Unadjusted.

Model 2: Adjusted for gender, age range, and BMI (overweight/obesity).

Model 3: Adjusted for gender, age range, BMI, and life risk factors (smoking, current drinking).

Model 4: Adjusted for gender, age range, life risk factors, and baseline health status (diabetes, hypertension, ischemic cardiomyopathy and hyperlipidemia).

**FIGURE 2:**
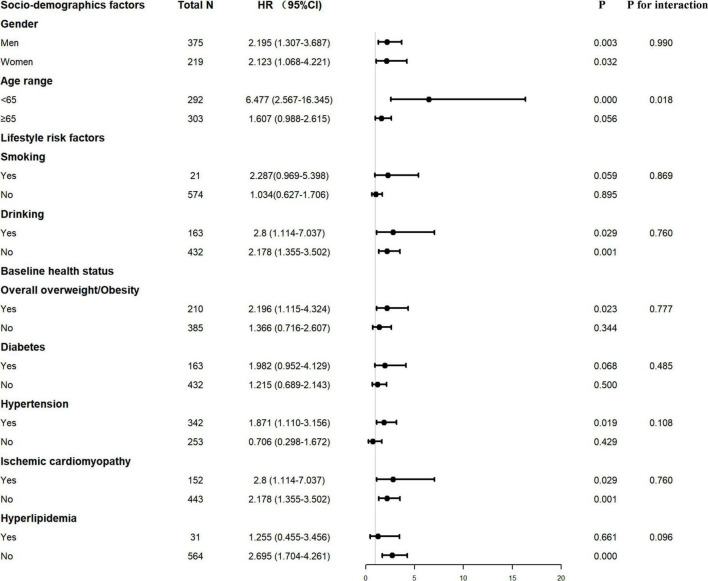
Interaction of confounding factors on the association between ΔPNI and major adverse cardiovascular event (MCE) risk. PNI, prognostic nutritional index.

**FIGURE 3:**
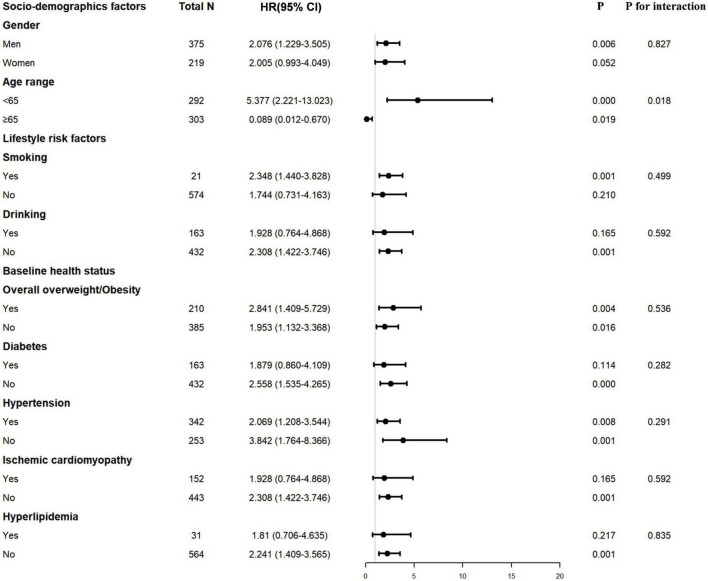
Interaction of confounding factors on the association between ΔGNRI and major adverse cardiovascular event (MCE) risk. GNRI, geriatric nutritional risk index.

Decreased PNI or GNRI scores were consistently associated with worse long-term clinical outcomes in each subgroup, including male or female, age ≥ 65 or < 65 years, with or without smoking and drinking history, with or without overall overweight/obesity, patients with or without hypertension or diabetes or ischemic cardiomyopathy or hyperlipidemia (Figures 2, 3).

**FIGURE 4:**
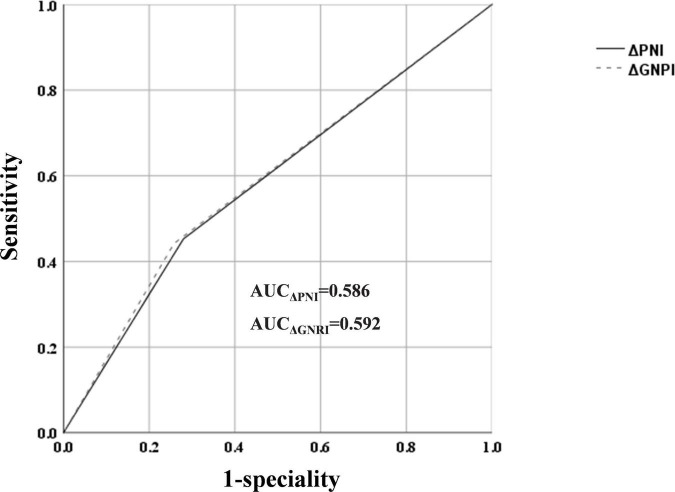
Receiver operating characteristic (ROC) curve result for ΔPNI and ΔGNRI.

ROC curve analysis revealed an AUC of 0.586 (sensitivity: 45.3%, specificity: 72%) for ΔPNI scores and AUC of 0.592 (sensitivity: 44.2%, specificity: 74.2%) for ΔGNRI scores, but their predictive abilities were not statistically different (Figures 3, 4).

## Discussion

The purpose of this study was to determine whether the changes in nutritional status, as measured by ΔPNI and ΔGNRI scores, was associated with MCEs in patients with PMI. The key finding of the present study was that a decrease in PNI or GNRI score during the follow-up was significantly and strongly associated with a higher risk of MCE. The changes in PNI and GNRI scores had similar predictive power for MCEs in our study population. To our knowledge, this is the first study to demonstrate the long-term predictive significance of the changes in the nutritional status evaluated by PNI and GNRI scores in patients with PMI.

Previous research had established that traditional cardiovascular risk variables such as age, renal dysfunction, male gender, a family history of coronary artery disease, heart failure, and diabetes are predictors of death following Pacemaker implantation (16). Additionally, more research had been conducted on the association between nutritional status and clinical outcomes (17, 18). In this study, we examined this association using the changes in PNI and GNRI scores during the follow-up. GNRI and PNI scores include the objective quantitative data of the patient. In the calculation of the GNRI, only the height, weight, and serum albumin data of the patient are required. Similarly, the calculation of the PNI needs only the serum albumin level and the lymphocyte count of the patient.

One study on gastrointestinal surgery (19) first described the as an integrated and multiparameter nutritional management paradigm in 1980. Since then, numerous studies had identified as an independent risk factor for poor clinic outcomes in a variety of conditions (20–22). The PNI score was suitable for evaluating distinct characteristics of two components and was straightforward to compute using affordable objective markers. The GNRI score was also an objective nutritional evaluation tool, calculated by a blood biomarker, height and weight, and several recent studies have demonstrated a link between this nutritional marker and cardiovascular risk in HF patients (12, 23). Considering the nutritional status was not still all the time, we chose the changes of the PNI and GNRI scores to evaluating their associations with MCEs.

A study enrolled patients undergoing cardiovascular surgery and found that a low PNI score was substantially linked with postoperative complications and survival and PNI score might be a valuable and reliable tool for assessing nutritional status before surgery, and it should be taken into account when determining the indication for and strategy for cardiovascular surgery (24). One study studied the association between PNI score and stable coronary artery disease and discovered that the PNI score was independently associated with higher MCE rates (25). Another research analyzed the link between acute HF and PNI score and found that PNI score was independently associated with cardiovascular death and total mortality (26). Yamada et al. (13) included 119 patients with a CRT-device and divided the patients based on whether their PNI score had increased at 6-month follow-up or not after the procedure. The PNI scores raised group had a lower occurrence of adverse events than the PNI scores decreased group in the Kaplan-Meier analysis. Newly published analysis (21) enrolling 141 patients with acute HF and performing multivariate analysis which displayed that PNI score in the improved group was independently associated with good outcomes. These outcomes were consistent with our studies. In addition to its use in the cardiovascular field, PNI score may be an effective index for predicting the rate of adverse events in other domains. A study enrolling patients with small hepatocellular carcinoma who had liver resection found that PNI score was an independent predictor of overall survival and recurrence-free survival in a multivariable study (27).

Geriatric nutritional risk index (GNRI) score was a validated nutritional assessment method screening nutritional conditions. A study investigated the association between GNRI score and stable coronary artery disease and reported that GNRI score ≤ 98 showed an increase in the incidences of cardiac death or non-fatal MI compared with GNRI score > 98 (28). Minamisawa et al. (29) found patients with low GNRI score had worse prognoses than those with high GNRI score in patients at risk for HF. A study on hemodialysis concluded the ΔGNRI score could predict all-cause, cardiovascular mortality and prove predictability for mortality (30). Another analysis studied the link of the changes in GNRI score and major adverse cardiac and cerebrovascular events in incident peritoneal dialysis patients, finding that patients with worsening or stationary GNRI score were at significantly greater risk for adverse events. These outcomes were well consistent with our results.

In this study, the area under the curve (AUC) of ΔPNI and ΔGNRI for determining MACs in patients with PMI was 0.586 and 0.592, respectively. However, these values may differ based on the patient population. Thus, the prognostic efficacy of ΔPNI and ΔGNRI in individuals with various cardiovascular disorders may not be constant.

In our study, we found the rates of MCE increased in both unadjusted and adjusted Cox modeling in decreased PNI and GNRI scores. Multivariate Cox hazard models demonstrated that both ΔPNI and ΔGNRI scores was independently associated with MCE after adjusting for risk factors. But we found a significant association between either ΔPNI or ΔGNRI score and age, which might be explained that the younger people have faster metabolisms and higher nutritional needs than the elderly. Once malnutrition occurred, it might be hit harder in this group.

Serum albumin, the most abundant plasma protein, was synthesized in the liver and secreted into the vascular space, where it was distributed throughout the body. It had traditionally been used to assess nutritional status and visceral protein synthesis function. Low levels of serum albumin had also been determined to be an independent risk factor for survival in patients after surgery (31, 32). Lymphocytes could be used to assess immunological nutritional status. Low lymphocytes meant poor immune status. Weight loss related with the development of cardiac cachexia was frequently connected with decreases in physical function and a worse prognosis. Thus, PNI score, combined serum albumin and lymphocyte could be a good index for screening nutritional status. The GNRI score, which was derived using serum albumin, ABW, and IBW while controlling for body edema, was an accurate index for assessing nutritional status.

## Limitation

Our study first evaluated the long-term predictive significance of ΔPNI and ΔGNRI score in patients with PMI. We also found that ΔPNI and ΔGNRI scores had similar predictive power in our study. However, our study had several limitations. First, as a single-center, observational study of a small patient cohort, unknown confounding factors might have affected the outcomes, regardless of analytical adjustments. Second, our population was limited to pacemaker implantation and could not be generalized to other populations. Third, considering the small population of the study, we did not perform a subgroup analysis between PNI or GNRI scores and HFH, MI, stroke, or death.

## Conclusion

Improved malnutrition risk assessed by the either ΔPNI or ΔGNRI were associated with increased cardiovascular events in PMI patients. The changes in PNI and GNRI scores had similar predictive power and might be useful for risk stratification of patients with pacing indications in clinical practice during the follow-up. By evaluating these two tools, we can forecast the probability of future adverse occurrences in patients during clinical work, which might help us better treat patients.

## Data availability statement

The raw data supporting the conclusions of this article will be made available by the authors, without undue reservation.

## Ethics statement

The studies involving human participants were reviewed and approved by the Institutional Review Board of the First Affiliated Hospital of Wenzhou Medical University (YS2022-306). The patients/participants provided their written informed consent to participate in this study.

## Author contributions

CC and XH designed the study, investigated, and revised the manuscript. KW and LL contributed to the investigation and drafted the manuscript. CPC and MW contributed to the investigation and prepared the figures. XH provided study supervision. All authors contributed to critical revision of the manuscript and approved its final version.
